# Regulation of CD163 Receptor in Patients with Abdominal Aortic Aneurysm and Associations with Antioxidant Enzymes HO-1 and NQO1

**DOI:** 10.3390/antiox12040947

**Published:** 2023-04-18

**Authors:** Bianca Hamann, Anna Klimova, Felicia Klotz, Frieda Frank, Christian Jänichen, Marvin Kapalla, Pamela Sabarstinski, Steffen Wolk, Henning Morawietz, David M. Poitz, Anja Hofmann, Christian Reeps

**Affiliations:** 1 Division of Vascular and Endovascular Surgery, Department of Visceral, Thoracic and Vascular Surgery, Faculty of Medicine and University Hospital Carl Gustav Carus, Technische Universität Dresden, D-01307 Dresden, Germany; 2 Core Unit Data Management and Analytics, National Center for Tumor Diseases Dresden (NCT/UCC), Faculty of Medicine and University Hospital Carl Gustav Carus, Technische Universität Dresden, D-01307 Dresden, Germany; 3 Division of Vascular Endothelium and Microcirculation, Department of Medicine III, University Hospital and Medical Faculty Carl Gustav Carus, Technische Universität Dresden, D-01307 Dresden, Germany; 4 Institute for Clinical Chemistry and Laboratory Medicine, University Hospital and Medical Faculty Carl Gustav Carus, Technische Universität Dresden, D-01307 Dresden, Germany

**Keywords:** abdominal aortic aneurysm, soluble CD163, bilirubin, antioxidative enzymes

## Abstract

Red blood cells are found within the abdominal aortic aneurysm (AAA), in the intraluminal thrombus (ILT), and in neovessels. Hemolysis promotes aortic degeneration, e.g., by heme-induced reactive oxygen species formation. To reduce its toxicity, hemoglobin is endocytosed by the CD163 receptor and heme is degraded by heme oxygenase-1 (HO-1). A soluble form (sCD163) is discussed as an inflammatory biomarker representing the activation of monocytes and macrophages. HO-1 and NAD(P)H quinone dehydrogenase 1 (NQO1) are antioxidant genes that are induced by the Nrf2 transcription factor, but their regulation in AAA is only poorly understood. The aim of the present study was to analyze linkages between CD163, Nrf2, HO-1, and NQO1 and to clarify if plasma sCD163 has diagnostic and risk stratification potential. Soluble CD163 was 1.3-fold (*p* = 0.015) higher in AAA compared to patients without arterial disease. The difference remained significant after adjusting for age and sex. sCD163 correlated with the thickness of the ILT (r_s_ = 0.26; *p* = 0.02) but not with the AAA diameter or volume. A high aneurysmal CD163 mRNA was connected to increases in NQO1, HMOX1, and Nrf2 mRNA. Further studies are needed to analyze the modulation of the CD163/HO-1/NQO1 pathway with the overall goal of minimizing the detrimental effects of hemolysis.

## 1. Introduction

Abdominal aortic aneurysms (AAA) are defined by a dilation of the aortic diameter >30 mm [1]. Surgical therapy is the treatment option of choice, and the decision largely relies on the maximum aortic diameter [1]. The discovery of novel biomarkers that diagnose AAA, predict disease progression, and/or identify fast-growing AAA has top priority in the field of research. The main pathomechanisms of AAA include oxidative stress, depletion of medial vascular smooth muscle cells (VSMC), inflammation, and proteolytic degradation of the extracellular matrix [1,2]. Approximately 75% of all AAA are covered by an intraluminal thrombus (ILT) that contains red blood cells (RBC), platelets, and hematopoietic stem cells embedded in a complex fibrin network [3]. RBC within the ILT or in medial and adventitial neovessels release hemoglobin (Hb upon hemolysis. The prosthetic heme group within the Hb strongly promotes reactive oxygen species (ROS) formation via the Fenton reaction [4,5]. To reduce its toxicity, free Hb is bound to haptoglobin (Hp), and the formed Hb-Hp complex is internalized by the CD163 receptor. Of importance, CD163 is exclusively found on the monocyte-macrophage cell lineage, and its expression is increased by anti-inflammatory mediators, suggesting an anti-inflammatory action. CD163 is considered a marker for a special subtype of macrophages, Mhem, that act in heme homeostasis and protect against free heme-induced toxicity [6,7]. The existence of protective CD163-positive macrophages has been shown in AAA walls [8].

A soluble form of CD163 (sCD163) was identified, and its release is stimulated under pathological conditions by, e.g., pro-inflammatory mediators or oxidative stress [9]. The cleavage of sCD163 is mediated by a metalloprotease-dependent shedding pathway [10]. Previous studies provided evidence of sCD163 being elevated in acute and chronic inflammatory disorders, reflecting tissue macrophage and monocyte activation [11]. However, the diagnostic and risk stratification potential of sCD163 in AAA has not been investigated so far.

After internalization, the degradation of heme from Hb is mediated by the heme oxygenase-1 (HO-1; encoded by the *HMOX1* gene) enzyme [7]. HO-1 catalyzes the breakdown of pro-oxidative heme, thereby forming carbon monoxide (CO), biliverdin, and ferrous iron ions (Fe^2+^) [12,13]. Biliverdin is converted to bilirubin, which is known for its various antioxidant properties [14]. Increased serum bilirubin has been shown to be associated with a lower risk for cardiovascular diseases [15]. An induction of HO-1 was shown in AAA and was associated with a lowering of oxidative stress [16].

The gene expression of HMOX1 is regulated by the transcription factor nuclear factor erythroid 2-related factor 2 (Nrf2; encoded by *Nfe2l2* gene). Nrf2 maintains cellular redox homeostasis by regulating the expression of antioxidant and detoxifying enzymes [17]. NAD(P)H-quinone oxidoreductase 1 (NQO1) is another Nrf2-regulated enzyme that catalyzes the detoxification of harmful arylating and oxidative quinones, thereby preventing ROS formation. The antioxidant capacity of NQO1 is attributed to its ability to maintain lipid soluble antioxidants in a reduced state and to protect membranes from lipid peroxidation [18]. Induction of NQO1 in vascular cells might be an adaptive mechanism against pro-inflammatory cascades and oxidative stress, thus making NQO1 an interesting therapeutic target [18]. However, nothing is known about the NQO1 expression and its associations with CD163 and Nrf2 in AAA.

## 2. Methods

### 2.1. Acquisition of Aortic Tissue

In total, 35 patients with AAA who underwent elective open surgical repair (eAAA) and 11 patients with ruptured AAA (rAAA) were included. Aortic tissue was collected intraoperatively from the left anterior aortic wall between March 2017 and August 2022. The eAAA group included 7 patients and the rAAA group 4 patients, respectively, from whom two different specimens of the same AAA sac were collected. Control samples were obtained from patients with arterial occlusive disease (AOD, n = 6) who underwent aortofemoral or aortobifemoral bypass surgery. The aorta was collected from the insertion side of the bypass. Control samples were free of histopathological aneurysmal dilatation. All specimens were collected within 10 min after removal, rinsed in 1xDPBS, cleaned from adjacent thrombus as well as blood clots, and immediately frozen in liquid nitrogen [16,19].

### 2.2. Study Design and Study Participants

The potential of sCD163 and bilirubin in diagnosing and predicting AAA was analyzed in a retroprospective, observational cohort study in electively treated patients with AAA as well as patients with varicose veins who served as controls. Patients were recruited within the Department of Visceral, Thoracic, and Vascular Surgery between January 2017 and February 2023. Male and female varicose patients were selected by >50 years of age and had no reported history of arterial disease [16]. Diagnostic criteria for varicose veins were venous reflux assessed by duplex ultrasound and signs of chronic venous incompetence according to the CEAP classification [20]. Patients with acute thrombophlebitis and known infectious diseases were excluded. Inclusion criteria for AAA were a diameter of the infrarenal aorta exceeding 40 mm, fast-growing AAA with more than 10 mm progress per year, or symptomatic AAA. Two patients received endovascular repair due to an iliac artery aneurysm but were also diagnosed with an aortic dilatation [19]. All subjects gave their informed consent for inclusion before they participated in the study. The study was conducted in accordance with the Declaration of Helsinki, and the protocol was approved by the Technische Universität (TU) Dresden (EK 151042017, 27 April 2017).

### 2.3. Outcome Variables, Confounders and Sample Size

Being diagnosed with either AAA or varicose vein was set as the dependent variable, and plasma sCD163 and serum bilirubin were set as the independent primary outcome variables. The absence or presence of AAA was measured categorically (1 = yes, 0 = no), while sCD163 and bilirubin were set as continuous variables. Age, smoking, sex, PAD, and statin intake were tested as potential confounders. Sample size/power calculation are not compulsory for observational studies [21]. Our study is exploratory, aiming to study the variability in biomarker values.

### 2.4. Clinical Variables

Serum low-density (LDL), high-density (HDL) lipoprotein, total cholesterol, triglycerides, non-fasting glucose, hemoglobin (Hb), bilirubin, leukocyte, and C-reactive protein (CRP) concentrations were determined in the Institute for Clinical Chemistry and Laboratory Medicine at the TU Dresden using standard laboratory methods. Due to the lack of data on blood chemistry or medical therapies, the number of patients varies in each group. Cardiovascular risk factors, comorbidities, and prescribed medical therapies were evaluated retrospectively. Hypertension, type 2 diabetes (T2D), heart failure (HF), peripheral artery disease (PAD), and carotid artery stenosis (CAS) were defined by a documented history of diagnosis or any treatment for these diseases. Coronary artery disease (CAD) was defined as any history of myocardial infarction, angina pectoris, or treatment for CAD. Smoking was assessed retrospectively and was defined as any history of smoking. Sex was self-reported.

The mean aortic diameter was assessed by computed tomography angiography (CTA) by measuring the distance from the outer adventitia to the outer adventitia by a single trained observer. ILT thickness was assessed in the arterial phase on CT scans following multiplanar reconstruction. The aorta was scanned in an axial position using a slice thickness of 1 mm. The thickness of the ILT was assessed at the largest distance between the inner surface of the lumen and the outer aortic wall. The AAA volume was measured by two trained people using the automatic segmentation model of the IMPAX EE R20 software (Agfa HealthCare, Mortsel, Belgium). The aorta was scanned with contrast media in the arterial phase in 1 mm sections. The outer wall of the aneurysm and the true lumen were selected manually every 6 mm in the transverse plane. The starting point of the measurement was defined as the cranial end of the aneurysm with a diameter greater than 30 mm. The measurement ended caudal to the dilation or at the aortic bifurcation. Non-recognized areas were cropped manually. The volume of AAA is given in cm^3^. The ILT was included in the total AAA volume measurement but was not specifically tagged for segmentation.

### 2.5. Blood Sampling and Enzyme-Linked Immunosorbent Assay (ELISA) for Determination of sCD163

Non-fasting venous blood was collected preoperatively in a 9 mL S-Monovette EDTA (Sarstedt, Nümbrecht, Germany) between 2017 and 2023. Blood samples were centrifuged at 2000× *g* for 10 min and plasma was stored in aliquots at −80 °C. Plasma sCD163 (ab274394, abcam, Cambridge, United Kingdom) was determined by enzyme-linked immunosorbent assay (ELISA) according to the manufacturer’s instructions. Samples were analyzed in duplicate using a dilution of 1:500.

### 2.6. RNA Isolation, cDNA Synthesis, and Quantitative Real-Time Polymerase Chain Reaction (qPCR)

Aortic tissue (30–50 mg) was homogenized in 1 mL TriFast (VWR, Darmstadt, Germany) using a Precellys 24 homogenizer (VWR, Darmstadt, Germany), and RNA was isolated according to the manufacturer’s instructions. Subsequently, an RNA clean and concentrator kit (R1016, Zymo Research, Freiburg im Breisgau, Germany) was used. An additional on-column DNase I digestion was performed to remove any remaining DNA. Reverse transcription of mRNA into cDNA was done using MultiScribe reverse transcriptase (Thermo Fisher Scientific, Darmstadt, Germany) with random hexamer primers according to the manufacturer’s instructions. Quantification of mRNA expression was done by real-time PCR with the GoTaq qPCR master mix (Promega, Walldorf, Germany) and the Step One Plus Real-Time PCR system (Thermo Fisher Scientific, Darmstadt, Germany). The analysis of the raw data was done with Step One Software version 2.3 (Thermo Fisher Scientific, Darmstadt, Germany), and the data are calculated as ΔCT values. The geometric mean of the 3 reference genes, ribosomal protein L32 (RPL32), TATA-box binding protein (TBP), and β-2-microglobulin (B2M), was used for cDNA content normalization. To ensure comparability of data, an internal control was included in each reverse transcription and qPCR. The mRNA expression was normalized to this control (=1) [16,19]. The efficiency of each primer pair was checked prior and was >90%. Primer sequences are given in Supplementary Table S1.

### 2.7. Statistical Analyses

Graph Pad Prism 9.0 (GraphPad Software, Inc., La Jolla, CA, USA) and R Stats Package (Boston, MA, USA) software were used for statistical analysis, and *p* ≤ 0.05 was considered significant. Significant outliers were tested by the Grubbs tests. Normality was tested by the D’Agostino and Pearson test. Comparison of more than two groups in Non-Gaussian distributed data was done by Kruskal–Wallis with Dunn’s multiple comparison test, and Gaussian distributed data by One-way ANOVA and Tukey’s post hoc test. All data are shown as box plots with individual values or as dot plots as indicated in the figure legend. Spearman’s correlational analysis was used for non-Gaussian-distributed data. Gaussian-distributed data were analyzed by Pearson’s correlation, and the respective correlation coefficient (r_S_/r_P_) was calculated. Differences in the distribution of cardiovascular risk factors and medical therapies were analyzed using the Chi-square test (χ^2^). The null hypothesis (H_0_) postulated that distributions of risk factors and medical therapies do not affect the outcome variable.

Weighted linear regression was used to identify medical therapies and cardiovascular risk factors with a potentially stronger or weaker effect on sCD163 and bilirubin. The factors that were identified in this way were further analyzed in a multiple linear regression model with soluble sCD163 (log transformed) or bilirubin, respectively, as an outcome variable. Furthermore, sCD163 and bilirubin were tested as responses to the prescription of different medical therapies and cardiovascular risk factors by multiple logistic regression. The odds ratios (ORs) represent the x-fold changes in sCD163, or bilirubin caused by the prescription of the indicated therapies or comorbidities. All estimates and ORs are presented on the >/<1 scale.

## 3. Results

### 3.1. Patients’ Characteristics for Quantification of Aortic mRNA Expression

Herein, electively treated and ruptured AAA as well as AOD controls were analyzed. The mean age differed between all groups, whereas rAAA (71.7 ± 5.9 y) had the highest age, followed by eAAA (65.0 ± 7.8 y) and AOD (55.5 ± 7.4 y). Patients with rAAA revealed the highest aortic diameter (87.78 ± 18.66 mm) compared to eAAA (61.74 ± 14.08 mm) and AOD (19.28 ± 4.16 mm) (*p* < 0.0001). A detailed description of patients is given in Table 1.

### 3.2. Aortic CD163 mRNA Expression and Linkage to the Antioxidant System

**Figure 1:**
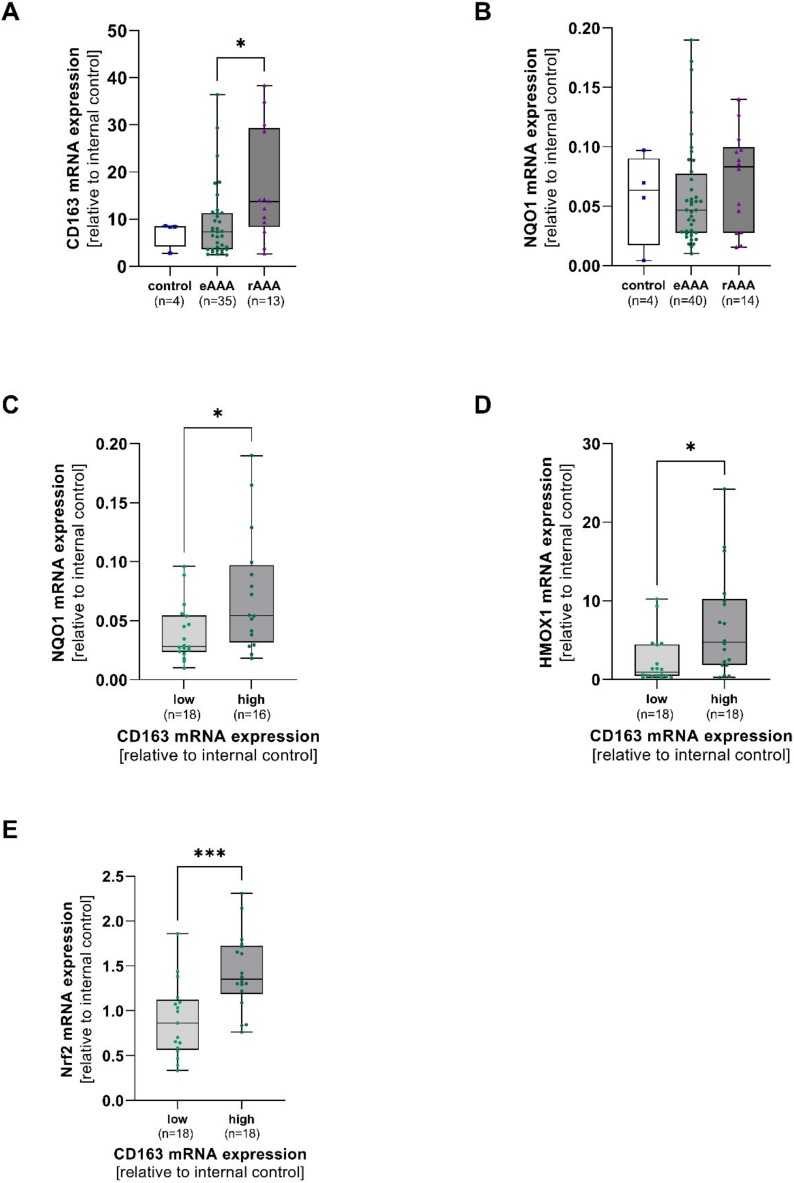
Linkage of aortic CD163 mRNA expression and antioxidant enzymes in AAA and corresponding controls. Aortic mRNA expression of CD163, NAD(P)H quinone dehydrogenase 1 (NQO1), heme oxygenase-1 (HMOX1), and nuclear factor erythroid 2-related factor 2 (Nrf2) was analyzed by qPCR. Data are presented in relation to an internal control (=1). (**A**), Expression of CD163, and (**B**), NQO1 mRNA in electively treated (eAAA), ruptured AAA (rAAA), and AOD controls. (**C**–**E**), CD163 mRNA was divided into low (light green) and high (dark green) depending on the median of the data set, and NQO1, HMOX1, and Nrf2 mRNA were grouped accordingly. Data on CD163 mRNA expression was not available for 2 AOD, 6 eAAA, and 2 rAAA patients. Data on NQO1 mRNA are missing for 2 AOD, 2 eAAA, and 1 rAAA patient. No Nrf2 mRNA expression data were available for 2 eAAA and 1 rAAA patient. **Statistics:** Outliers were identified by using Grubb’s test and were excluded from further analysis. Data shown as boxplots with individual values. (**A**,**B**) Kruskal-Wallis with Dunn’s post hoc test * *p* ≤ 0.05. (**C**,**D**) Mann-Whitney U test * *p* ≤ 0.05. (**E**) unpaired *t*-test, *** *p* ≤ 0.001.

Aortic mRNA expression of CD163, NQO1, Nrf2, and HMOX1 was analyzed in AOD, eAAA, and rAAA patients. Aortic CD163 mRNA expression turned out to be higher in rAAA when compared to eAAA (*p* = 0.040). No differences in NQO1 mRNA were observed between all groups (Figure 1A,B). CD163 mRNA was divided into low and high expression levels depending on the median of the data set. NQO1 (*p* = 0.03), HMOX1 (*p* = 0.010), and Nrf2 (*p* = 0.001) were increased in samples with high CD163 mRNA expression (Figure 1D,E). However, Nrf2 mRNA expression did not significantly differ between eAAA, rAAA, and AOD. Interestingly, NQO1 mRNA expression did not change, whereas HMOX1 mRNA expression increased with increasing Nrf2 mRNA expression (*p* = 0.04) (Supplementary Figure S1A–C).

### 3.3. Patients’ Characteristics for Assessment of sCD163 and Bilirubin Concentrations

To evaluate the potential of sCD163 as a biomarker for diagnosing and predicting AAA, sCD163 was measured in AAA and varicose vein controls. The AAA group comprised more males, and varicose patients were significantly younger ((median: 63.5, range 50.0–82.0 y) vs. 73.5 y, range: 53.0–89.0 y), *p* < 0.0001)). Both groups revealed differences in the prevalence of cardiovascular risk factors and the prescription of cardiometabolic pharmacotherapy (Table 2). No differences in the prevalence of other diseases, e.g., cancer, arthritis, asthma, pulmonary fibrosis, and chronic obstructive pulmonary disease, were observed between both groups (data not shown).

### 3.4. Diagnostic Value of sCD163 and Associations with Aortic Diameter, AAA Volume and Thickness of ILT

**Figure 2:**
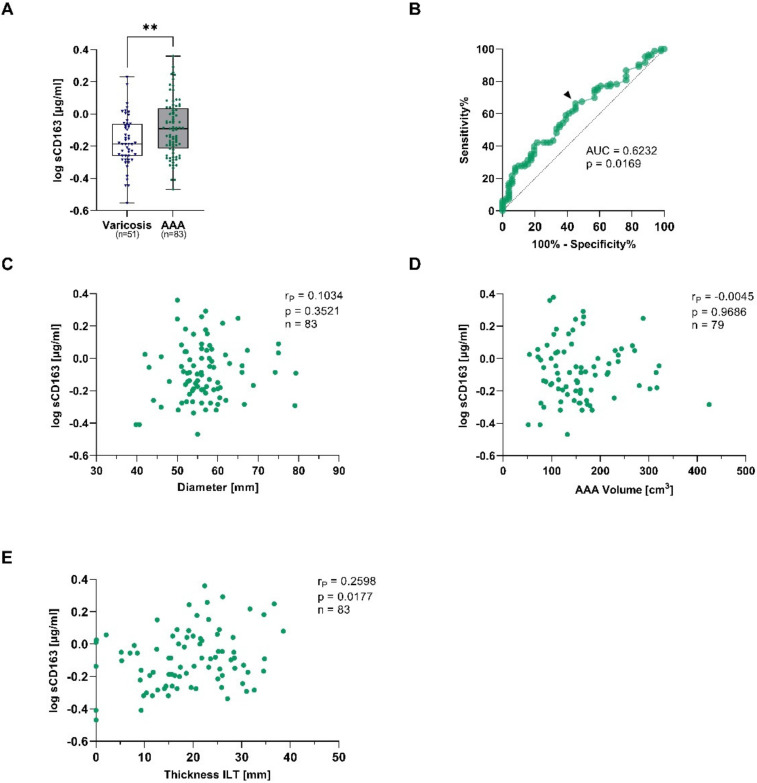
Plasma soluble CD163 concentrations and connections to AAA diameter, AAA volume, and thickness of the intraluminal thrombus (ILT) in AAA and corresponding controls. Plasma sCD163 was measured using ELISA, and and data were log transformed. (**A**), sCD163 concentrations were quantified in electively treated AAA patients (eAAA, green), and varicose controls (blue). (**B**), Receiver-operating characteristics (ROC) were used to determine the area under the curve (AUC) for obtaining the optimal cut-off value to discriminate AAA from controls with a high sensitivity. The black arrow indicates where the cut-off value was set. sCD163 concentrations were analyzed for correlations with (**C**), AAA diameter, (**D**), AAA volume and (**E**), thickness of the intraluminal thrombus (ILT). Data on sCD163 was not measurable in 2 varicose vein and 2 eAAA patients. In addition, no information on AAA volume was available for 4 eAAA patients. **Statistics:** Significant outliers were identified by Grubb’s outlier test and excluded from further analysis. The data are shown as (**A**), boxplots with individual values and were analyzed by unpaired *t*-test. ** *p* ≤ 0.01. (**C**–**E**), Pearson’s correlation coefficient (r_P_).

The soluble form of CD163 (sCD163) is released upon shedding from the membrane-bound form under pro-inflammatory conditions and therefore reflects monocyte and macrophage activation [22]. AAA patients had 1.3-fold higher sCD163 concentrations when compared to patients with venous vessel varicose (median: 0.81 vs. 0.65 µg/mL, *p* = 0.015) (Figure 2A). The observed increase was still present after adjusting for differences in age, sex, PAD, smoking, and statin treatment (*p*_adjusted_ = 0.008) (Table 3). In order to verify the diagnostic potential, a receiver-operating characteristic (ROC) analysis was performed. The area under the curve (AUC), which was used to assess the overall ability to discriminate between AAA patients and controls, was 0.623 (Figure 2B). To analyze the risk stratification potential, sCD163 was correlated with the AAA diameter, AAA volume, and the thickness of the ILT. Herein, sCD163 was not associated with the AAA diameter or volume, but concentrations increased with the thickness of the ILT (r_P_ = 0.260, *p* = 0.018) (Figure 2C–E).

To test the effects of cardiovascular risk factors and the prescription of pharmacological therapy on sCD163 concentrations in the AAA group, a weighted linear regression was performed. sCD163 was positively associated with HDL cholesterol, T2D treatment, triglycerides, and the prescription of β-blockers, whereas CCB, CAD, and PAD were linked with a lowering in sCD163 (Supplementary Table S2). For conducting a multivariate linear regression analysis, the above-named variables were chosen because they showed strong linkages with sCD163. After adjusting for HDL cholesterol, triglycerides, smoking, CAD, PAD, CCB, β-blockers, and T2D treatment, the increase in sCD163 with the thickness of the ILT tended to remain significant (β = 1.313, *p* = 0.064). Moreover, elevated sCD163 concentrations were associated with an increase in HDL cholesterol (β = 1.390, *p* = 0.034), triglycerides (β = 1.152, *p* = 0.017), the prescription of β-blockers (β = 1.313, *p* = 0.006), and, partly, with T2D treatment (β = 1.225, *p* = 0.055). In contrast, sCD163 concentrations tended to decrease with the prevalence of PAD (β = 0.820, *p* = 0.053) (Table 4).

### 3.5. Serum Bilirubin of AAA Patients and Associations with Aortic Diameter, AAA Volume and Thickness of ILT

Bilirubin, a secondary product of HO-1, was quantified in the serum of AAA and varicose control patients. Bilirubin concentrations were comparable between both groups (Supplementary Figure S3A). Interestingly, adjusting for age and sex revealed lower bilirubin concentrations in AAA (univariable analysis: OR = 0.978, *p* = 0.786; multivariable analysis: OR = 0.784, *p* = 0.019). The multivariate analysis revealed that bilirubin is strongly increased by age and male sex (Table 5). Next, correlations with morphological characteristics of the AAA were tested, and no associations with the AAA diameter (r_s_ = −0.113, *p* = 0.306), AAA volume (r_s_ = −0.167, *p* = 0.139), or thickness of the ILT (r_s_ = −0.151, *p* = 0.170) were found (Supplementary Figure S3B–D).

Further, a weighted linear regression was used to test the effects of cardiovascular risk factors and medical therapies on total bilirubin. Hemoglobin, insulin, diuretics, anticoagulation, and β-blockers were associated with higher bilirubin concentrations, whereas the prescription of ASA and ACE inhibitors, hypertension, triglycerides, and HDL cholesterol were associated with a lowering in bilirubin (Supplementary Table S3). Based on the effects of the weighted regression, a multivariate linear regression was used to test associations of bilirubin with the AAA diameter. No linkages between AAA diameter and bilirubin were found, also after adjusting to HDL cholesterol, triglycerides, hypertension, CAD, T2D, ACE, ARB, ASA, β-blockers, anticoagulation, diuretics, insulin, and T2D treatment. Nonetheless, an increase in serum bilirubin was associated with the prescription of diuretics (β = 1.300, *p* = 0.04) and insulin (β = 1.779, *p* = 0.010) (Supplementary Table S4).

## 4. Discussion

In the present study, we analyzed the regulation of the anti-inflammatory and detoxifying CD163 receptor along with the antioxidant, Nrf2-induced enzymes HO-1 and NQO1 in patients with AAA. Linkages between both systems were assessed with the overall aim of evaluating if the CD163-Nrf2/HO-1/NQO1 pathway is a novel target to treat inflammation and oxidative stress originating from intramural bleeding or the ILT in AAA. Finally, surrogate markers of these pathways were analyzed and tested for their diagnostic and risk stratification potential.

The soluble form of CD163 (sCD163) is cleaved from the membrane-bound form [22] by the action of the metalloprotease tumor necrosis factor-α–converting enzyme/ADAM metallopeptidase domain 17 (TACE/ADAM17) in monocytes and macrophages [23]. Herein, plasma sCD163 was measured in a retroprospective, observational study, and concentrations turned out to be higher in patients with AAA when compared to varicose vein patients with no history of arterial disease. The difference remained significant after adjusting for age, sex, smoking, PAD, and statin treatment. An elevation of sCD163 was shown in inflammatory [24,25,26,27] and atherosclerotic diseases [28,29,30]. Herein, sCD163 was lowered in patients with AAA and diagnosed with PAD. This is contrary to previous published studies in PAD [29] and could be due to the prescription of cardiometabolic therapies and their pleiotropic effects. Soluble CD163 concentrations are known to reflect the activity of monocytes and macrophages [30], confirming the inflammatory nature of AAA [31]. In this regard, it is known that monocytes in AAA have greater adhesion and transmigration potential [32].

The origin of sCD163 is not clarified yet and ranges from general macrophage activation, M2 macrophages, or the transition between M1 and M2 macrophages after the acute phase of injury [30]. Herein, sCD163 was not associated with the aortic diameter as the major predictor of AAA rupture or in the decision towards surgical resection. Classically activated macrophages are found in the aortic walls in late-stage AAA [33] but an increase in their number with AAA diameter has not been shown [34]. Another possible explanation is pharmacological therapies that might have affected CD163-expressing macrophages or the shedding of metalloproteases. It has been shown that glucocorticoids increase CD163 [35]. Macrophages in other tissues than the AAA might have contributed to the sCD163 concentrations. An inverse correlation between monocytic CD163 mRNA expression and sCD163 has been shown, which was not due to changes in the absolute number of monocytes, suggesting that activation state is more relevant [11]. These findings are partly supported by the inverse correlation between leukocytes and sCD163 in patients with AAA (see Supplementary Figure S2).

We furthermore quantified the AAA volume as another predictor of rupture risk and showed that sCD163 was not associated with the volume. Three-dimensional volume measurements consider the AAA lumen and the ILT and may therefore reflect changes in AAA morphology more precisely [36,37]. Of importance, we could demonstrate a clear positive relation between sCD163 and the thickness of the ILT for the first time. It has been shown that the ILT contains activated macrophages [38] and a release of CD163 from these cells into the circulation could be possible. This has been demonstrated already for other markers in AAA [39]. It is known that the ILT sequesters proteins rather than actively releasing them [40]. In addition, sCD163 has been identified in the conditioned media of AAA walls [8] and was increased in the conditioned media of hemorrhagic stenosing plaques [29].

Neovascularization has been observed in AAA [41] and the extravasation of erythrocytes with intravascular hemolysis might promote an increase in oxidative stress [42] and cause the shedding of CD163 [29]. After the internalization of hemoglobin by CD163, heme is degraded by HO-1 [43], leading to the formation of biliverdin, which is subsequently converted to the antioxidant bilirubin. In the present study, serum bilirubin was analyzed for the first time in patients with AAA. Bilirubin is a powerful ROS scavenger [44] and an anti-inflammatory molecule with immunomodulatory effects [14] but its in vivo relevance remains under debate. Bilirubin was comparable in AAA and unmatched varicose vein patients, but bilirubin was lower in AAA after adjusting for age and sex. However, patients in the AAA group were significantly older, and gradual increases in serum bilirubin have been shown with aging [45]. It was postulated that aging-induced bilirubin is caused by the reduction in red blood cells and the increase in aged erythrocytes [46]. In addition, the decline in bilirubin in cardiovascular diseases could be attributed to anemia [46]. Herein, circulating bilirubin was strongly affected by male sex, which has been previously demonstrated [47]. Thus, differences between AAA and varicose could be due to the higher percentage of elderly men within the AAA group.

The lowering in bilirubin tended to be associated with a higher AAA diameter and thickness of the ILT, although the data did not reach significance. Elevated serum bilirubin was inversely associated with coronary artery plaque burden [48], the risk for incidence of major adverse cardiac and cerebrovascular events [49], lower coronary flow reserve [50] or a reduced carotid intima-media thickness [51]. In summary, the lowering of bilirubin in age- and sex-adjusted AAA patients underlines the role of bilirubin in cardiovascular diseases and its therapeutic potential [14]. Modulation of HO-1-dependent bilirubin generation is one therapeutic possibility [52].

It is known that CD163 expression is increased in the aortic walls of patients with AAA [8] but nothing is known about the regulation in ruptured AAA. Herein, CD163 mRNA expression was higher in ruptured AAA when compared to electively treated patients. Similar findings were demonstrated in symptomatic carotid artery stenosis and in plaques with a high vulnerability index [53]. The increase in ruptured AAA could be due to higher hemorrhage, as has been shown for intraplaque hemorrhage [54]. Analysis of ruptured AAA revealed an increase in genes involved in angiogenic responses [55], suggesting that neovascularization and subsequent hemolysis might have contributed to the increase in CD163 mRNA expression.

In the present study, associations between CD163 and expression of the antioxidant enzymes HO-1 and NQO1 in AAA were assessed for the first time. We proposed a mechanism of hemolysis in microvessels of the aortic wall and increased CD163-mediated uptake of hemoglobin-haptoglobin accompanied by an elevation in Nrf2 and in its target genes NQO1 and HMOX1. We could show for the first time that AAA walls with high CD163 mRNA expression had increased NQO1, Nrf2, and HMOX1 mRNA expression, reflecting that this pathway would be an interesting target. However, NQO1 mRNA expression was similar between electively treated and ruptured AAA, and expression was not linked to Nrf2 mRNA, although NQO1 is an Nrf2-target gene [56]. Analyzing Nrf2 translocation into the nucleus or its cellular localization would have been more appropriate [57] than analyzing mRNA expression. Furthermore, NQO1 expression could be controlled by the methylation status of the promoter [58] or the aromatic hydrocarbon (Ah) receptor [59]. NQO1 mRNA expression in the AAA group showed a strong scatter of the data, which could be due to the strong and rapid increase under stress conditions [59].

## 5. Conclusions

In conclusion, the present study shows that the soluble form of CD163 is higher in patients with AAA, underlining the diagnostic potential. Plasma sCD163 correlated with the thickness of the ILT but not with the AAA diameter or the AAA volume, thus showing that sCD163 may be related to the biological activities of different cells within the ILT. Furthermore, the present study showed for the first time that serum bilirubin concentrations are lower in age- and sex-matched patients with AAA and tend to be inversely correlated with the AAA diameter, AAA volume, and thickness of the ILT. This supports the hypothesis that increasing systemic or local bilirubin concentrations could be beneficial for patients with AAA. Finally, we could demonstrate for the first time that CD163 mRNA and the antioxidant enzymes HO-1 and NQO1 are linked in the aortic walls of patients with AAA. Further in vitro studies in macrophages are needed to assess the modulation of the CD163-Nrf2/HO-1/NQO1 pathway with the overall goal of minimizing detrimental effects of the ILT or intramural bleeding.

## 6. Limitations

The present study is an observational, descriptive study in a rather small cohort of patients and controls. Herein, varicose vein patients were used as a referral group. Their limitation is the rather low number of included patients and their different risk factor profiles. A low number of patients in combination with a high number of confounders could have affected the present findings. Analyzing sCD163 in risk factor-adjusted patients, e.g., in a case-control study, would have been one possibility. Establishing a causal relationship between tissue CD163 expression and circulating sCD163 requires studies in macrophages or preclinical AAA models. Analyzing CD163 protein expression and its localization within the AAA wall would have given more information on the involved areas within the aortic walls. Finally, we are not able to obtain information on HO-1, NQO1, or Nrf2 protein expression or their localization within the different layers of the aortic wall.

## Figures and Tables

**Table 1:** Clinical characteristics of patients with abdominal aortic aneurysm (AAA) and arterial occlusive disease (AOD) for analysis of aortic mRNA expressions.

Aortic mRNA Expression Study					
	AOD	eAAA	rAAA	*p*-Value	χ^2^
Baseline Demographics					
n included	6	35	11		
Age, years, mean ± SD, n	55.5 ± 7.47 6	64.80 ± 7.26 35	72.64 ± 6.30 11	0.0127 (AOD vs. eAAA) <0.0001 (AOD vs. rAAA) 0.0068 (eAAA vs. rAAA)	
Sex, male:female, % male, n	4:2, 67 6	32:3, 91 35	8:3, 73 11	0.14	3.93
Aortic diameter, mm, mean ± SD, n	19.28 ± 4.16 6	62.20 ± 14.09 33	82.89 ± 17.97 10	<0.0001 (AOD vs. eAAA) <0.0001 (AOD vs. rAAA) 0.0006 (eAAA vs. rAAA)	
Thickness ILT, mm, median with range, n	n.d.	16.70 (0.00–52.20) 31	30.60 (19.70–44.70) 9	0.001	
**Hematology**					
Hemoglobin, mmol/L, mean ± SD, n	7.52 ± 1.27 6	8.37 ± 1.43 35	7.35 ± 1.68 10	0.10	
Reference values 8.60–12.10 mmol/L					
Leukocytes, GPt/L mean ± SD, n	12.31 ± 4.10 6	8.00 ± 2.14 35	12.56 ± 4.91 10	0.0075 (AOD vs. eAAA) 0.0004 (eAAA vs. rAAA)	
Reference values 3.8–9.8 GPt/L					
**Cardiovascular risk factors**					
LDL cholesterol, mmol/L, median with range, n	1.47 (1.26–1.65) 4	2.52 (0.89–6.16) 21	n.d.	0.08	
Reference values < 1.40 mmol/L for people with very-high risk					
HDL cholesterol, mmol/L, mean ± SD, n	1.20 ± 0.28 4	1.18 ± 0.33 21	n.d.	0.94	
Reference values > 0.90 mmol/L for men and >1.10 mmol/L for women					
Total cholesterol, mmol/L, mean ± SD, n	2.97 ± 0.08 4	4.16 ± 1.34 21	n.d.	0.15	
Reference values < 4.00 mmol/L					
Triglycerides, mmol/L, mean ± SD, n	1.44 ± 0.39 5	1.91 ± 0.94 28	n.d.	0.28	
Reference values 0.35–1.70 mmol/L					
Blood glucose, mmol/L, mean ± SD, n	2.97 ± 0.08 4	5.55 ± 0.99 22	n.d.	0.0002	
Reference values only for fasting glucose					
CRP, mg/L, median with range, n	4.05 (1.20–18.00) 6	3.25 (0.50–21.00) 35	6.40 (0.50–65.00) 9	0.44	
Reference values < 5.0 mg/L					
Smoking, yes:no, %, n	5:1, 83 6	22:13, 63 35	4:7, 36 11	0.13	4.03
Hypertension, yes:no, %, n	4:2, 67 6	29:6, 83 35	10:1, 91 11	0.45	1.60
CAD, yes:no, %, n	3:3, 50 6	9:26, 26 35	4:7, 36 11	0.44	1.62
HF, yes:no, %, n	3:3, 50 6	7:28, 20 35	2:9, 18 11	0.25	2.79
CAS, yes:no, %, n	0:6, 0 6	1:34, 3 35	1:10, 9 11	0.56	1.15
PAD, yes:no, %, n	6:0, 100 6	8:27, 23 35	2:9, 18 11	0.0005	15.35
T2D, yes:no—%, n	1:5, 17 6	5:30, 12 35	2:9, 20 11	0.94	0.11
BMI, kg/m^2^, mean ± SD, n	25.28 ± 6.40 6	27.02 ± 4.17 35	29.09 ± 5.76 10	0.29	
**Medical therapies**					
Statins, yes:no, %, n	5:1, 83 6	21:14, 60 35	6:4, 60 10	0.54	1.23
ACE inhibitors, yes:no, %, n	0:6, 0 6	15:20, 43 35	4:6, 40 10	0.13	4.07
ARB, yes:no, %, n	2:4, 33 6	12:23, 34 35	3:7, 30 10	0.97	0.06
CCB, yes:no, %, n	0:6, 0 6	15:20, 43 35	4:6, 40 10	0.13	4.07
ASA, yes:no, %, n	6:0, 100 6	19:16, 54 35	6:4, 60 10	0.11	4.49
β-blocker, yes:no, %, n	2:4, 33 6	14:21, 40 35	4:6, 40 10	0.95	0.10
Anticoagulation, yes:no, %, n	2:4, 33 6	6:29, 17 35	2:8, 20 10	0.65	0.85
Antiplatelet, yes:no, %, n	1:5, 17 6	2:33, 6 35	2:8, 20 10	0.34	2.16
Diuretics, yes:no, %, n	0:6, 0 6	11:24, 31 35	4:6, 40 10	0.21	3.11
T2D treatment, yes:no, %, n	1:5, 17 6	4:31, 11 35	1:9, 10 10	0.92	0.17
Insulin, yes:no, %, n	1:5, 17 6	2:33, 6 35	0:10, 0 10	0.39	1.89

Aortic tissues were obtained from patients undergoing elective open repair (eAAA), open repair due to rupture (rAAA), or surgery because of arterial occlusive disease (AOD). Due to the emergency surgery, blood lipids were not quantified in rAAA. Further, for some patients, data on medical therapies and risk factors were not available in the medical reports, and the number of patients (n) varies. LDL, HDL, and total cholesterol were missing for 2 AOD and 14 eAAA patients; glucose for 2 AOD and 13 eAAA; and triglycerides for 1 AOD and 7 eAAA patients. Laboratory parameters were not available for all rAAA patients except CRP, which was only missing for 2 patients, and hemoglobin and leukocytes, which were missing for 1 patient. Furthermore, information about comorbidities and treatment were not given for 1 rAAA patient. **Statistics:** All data are presented as median with minimum and maximum (range) or as mean with standard deviation, depending on normality testing. The comparison of groups was done by the Mann-Whitney U test, Kruskal-Wallis, and Dunn’s post hoc test, an unpaired *t*-test, or a one-way ANOVA with Tukey’s post hoc test. The prevalence of risk factors and medical therapy were analyzed by the Chi-square (χ^2^) test. Treatment of type 2 diabetes (T2D) includes patients prescribed biguanides, sodium-glucose co-transporter-2 (SGLT2) inhibitors, glucagon-like peptide-1 receptor agonists (GLP-1-RA)/glucagon-like-peptide-1 receptor antagonists, dipeptidyl peptidase 4 (DPP-4)-inhibitors, or sulfonylureas. **Abbreviations:** AAA-abdominal aortic aneurysm; ACE–angiotensin-converting enzyme; AOD–arterial occlusive disease; ARB–angiotensin II receptor blocker; ASA–acetylsalicylic acid; BMI–body mass index; CAD–coronary artery disease; CAS–carotid artery stenosis; CCB–calcium channel blocker; CRP–C-reactive protein; HDL–high-density lipoprotein; HF–heart failure; LDL–low-density lipoprotein; ILT–intraluminal thrombus; PAD–peripheral artery disease; SD–standard deviation; T2D–type 2 diabetes mellitus.

**Table 2:** Clinical characteristics in patients with abdominal aortic aneurysm (AAA) and venous vessel varicose for the analysis of serum and plasma concentrations.

Retrospective, Observational Study				
	Varicosis	AAA	*p*-Value	χ^2^
Baseline Demographics				
n included	54	86		
Age, years, median with range, n	63.50 (50.00–82.99) 54	73.50 (53.00–89.00) 86	<0.0001	
Sex, male:female, % male, n	27:27, 50 54	82:4, 95 86	<0.0001	39.57
**Hematology**				
Hemoglobin, mmol/L, mean ± SD, n	8.88 ± 0.72 50	8.89 ± 1.04 86	0.97	
Reference values 8.60–12.10 mmol/L				
Leukocytes, GPt/L, mean ± SD, n	6.48 ± 1.50 50	7.54 ± 1.85 86	0.0007	
Reference values 3.8–9.8 GPt/L				
**Cardiovascular risk factors**				
LDL cholesterol, mmol/L, median with range, n	3.33 (0.85–5.62) 50	2.21 (0.71–5.21) 82	<0.0001	
Reference values < 1.40 mmol/L for people with very-high risk				
HDL cholesterol, mmol/L, median with range, n	1.64 (1.01–2.77) 49	1.26 (0.68–2.15) 83	<0.0001	
Reference values > 0.90 mmol/L for men and >1.10 mmol/L for women				
Total cholesterol, mmol/L, median with range, n	5.40 (2.12–7.74) 49	3.97 (2.05–6.46) 82	<0.0001	
Reference values < 4.00 mmol/L				
Triglycerides, mmol/L, median with range, n	1.28 (0.43–3.88) 50	1.48 (0.52–4.50) 83	0.24	
Reference values 0.35–1.70 mmol/L				
Blood glucose, mmol/L, median with range, n	5.21 (4.08–8.59) 49	5.51 (2.93–13.73) 85	0.06	
Reference value only for fasting glucose possible				
CRP, mg/L, median with range, n	1.30 (0.30–6.60) 49	2.40 (0.50–62.30 85	0.0003	
Reference values < 5.0 mg/L				
Smoking, yes:no, %, n	6:47, 11 53	45:41, 52 86	<0.0001	23.74
Hypertension, yes:no, %, n	27:26, 51 53	73:13, 85 86	<0.0001	18.71
CAD, yes:no, %, n	2:51, 4 53	32:54, 37 86	<0.0001	19.84
HF, yes:no, %, n	27:26, 51 53	73:13, 85 86	<0.0001	18.71
CAS, yes:no, %, n	0:53, 0 53	9:77, 10 86	0.02	5.93
PAD, yes:no, %, n	0:53, 0 53	23:63, 27 86	<0.0001	4.12
T2D, yes:no, %, n	3:50, 6 53	23:63, 27 86	0.005	7.97
Renal failure, yes:no, %, n	0:53, 0 53	11:75, 86 86	0.007	7.36
BMI, kg/m^2^, median with range, n	27.10 (16.60–40.70) 54	27.40 (18.81–37.13) 85	0.55	
**Medical therapies**				
Statins, yes:no, %, n	11:42, 21 53	66:20, 77 86	<0.0001	41.60
ACE inhibitors, yes:no, %, n	10:43, 19 53	27:59, 31 86	0.11	2.64
ARB, yes:no, %, n	14:39, 26 53	39:47, 74 86	0.03	4.98
CCB, yes:no, %, n	11:42, 21 53	35:51, 41 86	0.02	5.89
ASA, yes:no, %, n	3:50, 6 53	58:28, 67 86	<0.0001	7.13
β-blocker, yes:no, %, n	15:38, 28 53	43:43, 50 86	0.012	2.52
Anticoagulation, yes:no, %, n	17:36, 59 53	12:74, 14 86	0.011	2.55
Antiplatelet, yes:no, %, n	0:53, 0 53	9:77, 10 86	0.02	2.44
Diuretics, yes:no, %, n	12:41, 23 53	36:50, 42 86	0.021	2.32
T2D treatment, yes:no, %, n	2:51, 4 53	17:69, 20 86	0.008	7.11
Insulin, yes:no, %, n	1:52, 2 53	5:81, 6 86	0.27	1.11

Blood was collected preoperatively in the non-fasting state. Data on medical therapies and risk factors was not available for some patients, and the number of patients varies. Hemoglobin, leucocytes, LDL cholesterol, and triglyceride concentrations were not measured in 4 varicose vein patients. In addition, HDL and total cholesterol, glucose, and CRP were not available for 5 varicose patients, respectively. In eAAA patients, LDL and total cholesterol were not analyzed in 4 patients, HDL and triglycerides in 3 patients, and glucose and CRP in 1 patient, respectively. Data for comorbidities and medical therapy were not documented in 1 varicose vein patient. **Statistics:** All data are presented as median with minimum and maximum (range) or as mean with standard deviation, depending on normality testing. The comparison of groups was done using Mann-Whitney U, Kruskal-Wallis with Dunn’s post hoc test, an unpaired *t*-test or a one-way ANOVA with Tukey’s post hoc test. The prevalence of risk factors and medical therapy was analyzed by Chi-square (χ^2^). Treatment of type 2 diabetes (T2D) includes patients prescribed biguanides, sodium-glucose co-transporter-2 (SGLT2) inhibitors, glucagon-like peptide-1 receptor agonists (GLP-1-RA)/glucagon-like-peptide-1 receptor antagonists, dipeptidyl peptidase 4 (DPP-4)-inhibitors, and sulfonylureas. **Abbreviations:** AAA–abdominal aortic aneurysm, ACE–angiotensin-converting enzyme, AOD–arterial occlusive disease, ARB–angiotensin II receptor blocker, ASA–acetylsalicylic acid, BMI–body mass index, CAD–coronary artery disease, CAS–carotid artery stenosis, CCB–calcium channel blocker, CRP–C-reactive protein, HDL–high-density lipoprotein, HF–heart failure, LDL–low-density lipoprotein, ILT–intraluminal thrombus, PAD–peripheral artery disease, SD–standard deviation, T2D–type 2 diabetes mellitus.

**Table 3:** Plasma sCD163 concentrations and associations with AAA disease, age, sex, PAD and prescription of statins.

	Univariable Analysis			Multivariate Analysis		
Variable	OR	CI	*p*-Value	OR	CI	*p*-Value
Group (ref = varicose vein)	1.191	[1.036, 1.370]	0.015	1.336	[1.082, 1.649]	0.008
Age	1.010	[1.003, 1.016]	0.006	1.008	[1.000, 1.015]	0.054
Sex (ref = female)	0.976	[0.826, 1.154]	0.780	0.922	[0.758, 1.121]	0.415
PAD (ref = none)	0.922	[0.767, 1.110]	0.394	0.835	[0.692, 1.006]	0.060
Smoking (ref = none)	1.005	[0.869, 1.161]	0.951	0.977	[0.837, 1.141]	0.769
Statin (ref = none)	1.036	[0.902, 1.190]	0.615	0.914	[0.780, 1.071]	0.269

Plasma sCD163 was quantified by ELISA, and the data were log transformed. Age, sex, PAD, smoking, and prescription of statins were tested as confounding variables. Data were analyzed by multivariate logistic regression using sCD163 as an outcome variable, assuming that changes in sCD163 are due to AAA disease and not the cause. The odds ratio (ORs) refers to the relative increase or decrease in sCD163 when comparing the AAA group with varicose veins (ref = varicose veins). The ORs for medical therapies show the increase (>1) or decrease (<1) when the patient received the indicated therapy or had the diagnosed risk factors as compared to those without (ref = none). Univariable values were obtained by pairwise comparison of each variable listed in the table with sCD163 as the outcome variable. The data were analyzed by holding the effects of the other cardiometabolic therapies/risk factors constant and assuming high or low sCD163 concentrations. **Abbreviations:** CI, confidence interval; PAD, peripheral artery disease; OR, Odds Ratio.

**Table 4:** Plasma sCD163 concentrations and associations with the AAA diameter.

Variable	Estimate	CI	*p*-Value
AAA diameter	0.999	[0.985, 1.012]	0.832
Thickness ILT	1.010	[1.000, 1.021]	0.064
HDL cholesterol	1.390	[1.031, 1.875]	0.034
Triglycerides	1.152	[1.029, 1.291]	0.017
Smoking (ref = none)	0.929	[0.784, 1.102]	0.402
CAD (ref = none)	0.869	[0.716, 1.055]	0.160
PAD (ref = none)	0.820	[0.673, 0.999]	0.053
T2D treatment (ref = none)	1.225	[0.999, 1.501]	0.055
CCB (ref = none)	0.863	[0.718, 1.037]	0.120
β-blocker (ref = none)	1.313	[1.087, 1.586]	0.006

Plasma sCD163 was measured using ELISA, and the data were log transformed. sCD163 was set as the outcome variable, and effects of AAA diameter, thickness of ILT, HDL cholesterol, triglycerides, smoking, CAD, PAD, T2D treatment, CCB, and β-blockers were analyzed by multivariate linear regression. Estimates show the increase or decrease in sCD163 when the patient has the indicated disease or receives the respective medical therapy (ref = none). For AAA diameter, thickness of ILT, HDL cholesterol, and triglyceride estimates, refer to the increase or decrease per one unit. Treatment of type 2 diabetes (T2D) includes patients prescribed biguanides, sodium-glucose co-transporter-2 (SGLT2) inhibitors, glucagon-like peptide-1 receptor agonists (GLP-1-RA)/glucagon-like-peptide-1 receptor antagonists, dipeptidyl peptidase 4 (DPP-4) inhibitors and sulfonylureas. **Abbreviations:** AAA–abdominal aortic aneurysm, CAD–coronary artery disease, CCB–calcium channel blockers, CI–confidence interval, HDL–high-density lipoprotein, ILT–intraluminal thrombus, PAD–peripheral artery disease, T2D–type 2 diabetes mellitus.

**Table 5:** Serum bilirubin concentrations and associations with AAA disease, age, and sex.

	Univariable Analysis			Multivariate Analysis		
Variable	OR	CI	*p*-Value	OR	CI	*p*-Value
Group (ref = varicose vein)	0.978	[0.834, 1.147]	0.786	0.784	[0.642, 0.958]	0.019
Age	1.007	[1.000, 1.015]	0.058	1.012	[1.004, 1.020]	0.005
Sex (ref = female)	1.154	[0.957, 1.391]	0.135	1.350	[1.083, 1.684]	0.009

Serum bilirubin was quantified using standard laboratory assays at the Institute for Clinical Chemistry and Laboratory Medicine at the TU Dresden. Age and sex were tested as confounding variables. Data were analyzed by multivariate logistic regression using bilirubin as an outcome variable, assuming that changes in bilirubin are due to AAA disease and not the cause. The odds ratio (OR) refers to the relative increase or decrease in bilirubin when comparing the AAA group with varicose veins (ref = varicose veins). The ORs for medical therapies show the increase (>1) or decrease (<1) when the patient received the indicated therapy or had the diagnosed risk factors as compared to those without (ref = none). Univariable values were obtained by pairwise comparison of each variable listed in the table with bilirubin as the outcome variable. The data were analyzed by holding the effects of the other cardiometabolic therapies/risk factors constant and assuming high or low bilirubin. **Abbreviations:** CI, confidence interval; OR, Odds Ratio.

## Data Availability

Data is contained within the article.

## Supplementary Materials

The following supporting information can be downloaded at: https://www.mdpi.com/article/10.3390/antiox12040947/s1, Figure S1: Linkage of aortic mRNA expression of NAD(P)H quinone dehydrogenase 1 (NQO1), heme oxygenase-1 (HMOX1) and nuclear factor erythroid 2-related factor 2 (Nrf2) in AAA; Figure S2: Correlational analysis of sCD163 and bilirubin with leukocytes and hemoglobin in patients with AAA; Figure S3: Serum bilirubin concentrations and correlations with AAA diameter, AAA volume and thickness of the intraluminal thrombus (ILT) in AAA and controls. Table S1: Primersequences; Table S2: Plasma sCD163, cardiovascular risk factors and medical therapy in AAA patients; Table S3: Serum bilirubin concentrations, cardiovascular risk factors and medical treatment in AAA; Table S4: Serum bilirubin concentrations and associations with the AAA diameter.

## Nonstandard Abbreviations and Acronyms

AAA–abdominal aortic aneurysm, ACE–angiotensin-converting enzyme, AOD–arterial occlusive disease, ARB–angiotensin II receptor blocker, ASA–acetylsalicylic acid, AUC–area under the curve, B2M–β-2-microglobulin, BMI–body mass index, CAD–coronary artery disease, CAS–carotid artery stenosis, CCB–calcium channel blocker, CRP–C-reactive protein, CT–computed tomography, eAAA–electively treated AAA, Hb–hemoglobin, HDL–high-density lipoprotein, HF–heart failure, HMOX1/HO-1–heme oxygenase-1, Hp–haptoglobin, ILT–intraluminal thrombus, LDL–low-density lipoprotein, NQO1–NAD(P)H dehydrogenase quinone 1, Nrf2–nuclear factor erythroid 2-related factor 2, PAD–peripheral artery disease, RPL32–ribosomal protein 32, rAAA–ruptured AAA, RBC–red blood cells, ROC–receiver-operating characteristic, ROS–reactive oxygen species, sCD163–soluble CD163, SD–standard deviation, T2D–type II diabetes, TBP–TATA-box binding protein, VSMC–vascular smooth muscle cells.
