# Supplementary material for: Regulation of CD163 Receptor in Patients with Abdominal Aortic Aneurysm and Associations with Antioxidant Enzymes HO-1 and NQO1
Source: Antioxidants (Basel). 2023 Apr 18;12(4):947. doi: 10.3390/antiox12040947 (PMC10135987; doi:10.3390/antiox12040947)
Supplement: Supplementary file 1 [file antioxidants-12-00947-s001.zip › antioxidants-2319316-supplementary.pdf]

Supplementary Figures and Tables

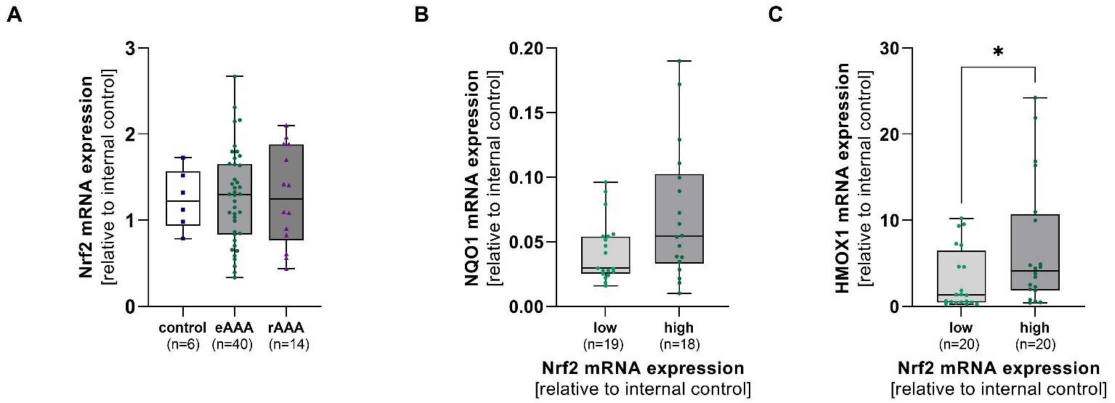

Supplementary Figure S1. Linkage of aortic mRNA expression of NAD(P)H quinone dehydrogenase 1 (NQO1), heme oxygenase-1 (HMOX1), and nuclear factor erythroid 2-related factor 2 (Nrf2) in AAA.

Nrf2, NQO1, and HMOX1 mRNA expression was analyzed by qPCR, and the data are presented in relation to an internal control (=1). **A,** Expression of Nrf2 in electively treated AAA (eAAA), ruptured AAA (rAAA) and AOD controls. Nrf2 mRNA expression was divided into low (light green) and high (dark green) expression depending on the median of the data. **B,** NQO1, and **C,** HMOX1 mRNA expression was grouped accordingly. Data for NQO1 mRNA expression was not available for 2 eAAA and 1 rAAA patients, respectively. **Statistics:** Data are shown as boxplots with individual values. Outliers were identified using Grubb´s outlier test and excluded from further analysis. **A**, a one-way ANOVA with Tukey´s *post hoc* test; **B,** unpaired t-test; and **C,** Mann-Whitney U test *$p \leq 0.05$.

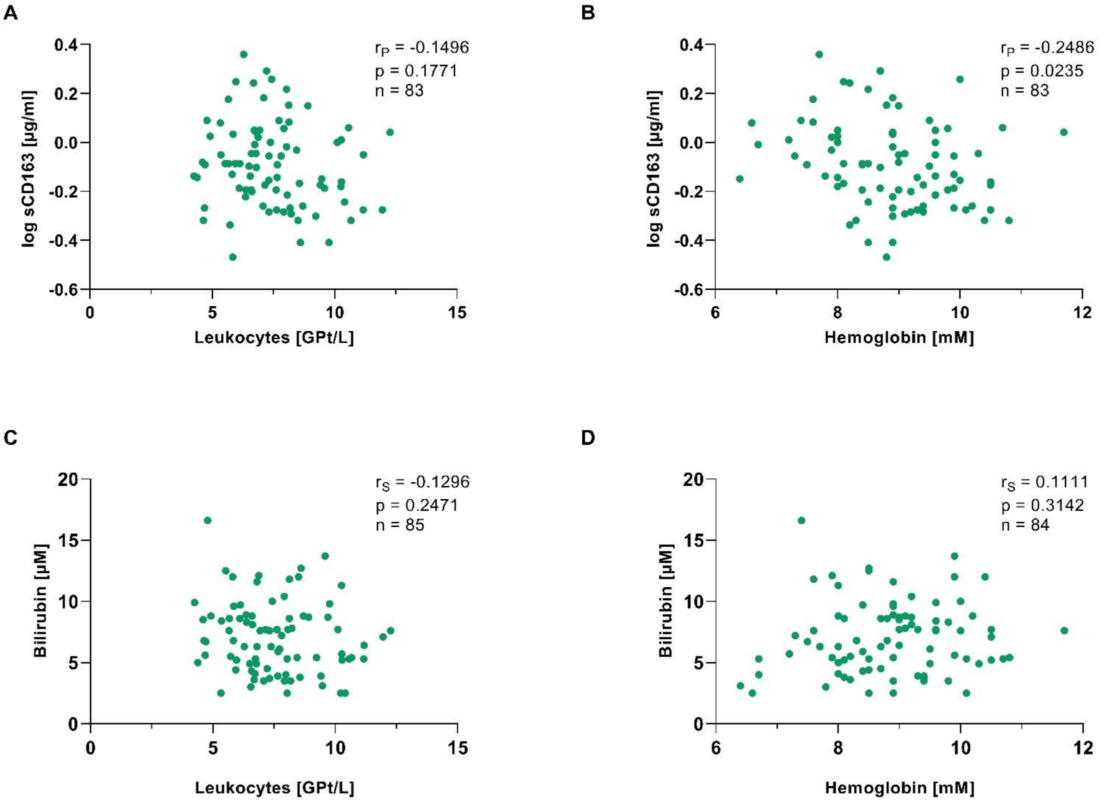

Supplementary Figure S2. Correlational analysis of sCD163 and bilirubin with leukocytes and hemoglobin in patients with AAA.

Plasma sCD163 was measured by ELISA, and the data were log transformed. Total bilirubin, leukocytes, and hemoglobin were measured in serum using standard laboratory methods at the Institute for Clinical Chemistry and Laboratory Medicine at the TU Dresden. Data on sCD163 and bilirubin were not available for 2 and 1 patient, respectively. **Statistics:** Pearson's correlation (rp) coefficient between sCD163 and **A,** leukocytes, and **B,** hemoglobin. Spearman's correlation coefficient (rs) between bilirubin and **C,** leukocytes, and **D,** hemoglobin. Outliers were identified using Grubb´s outlier test and were excluded from further analysis.

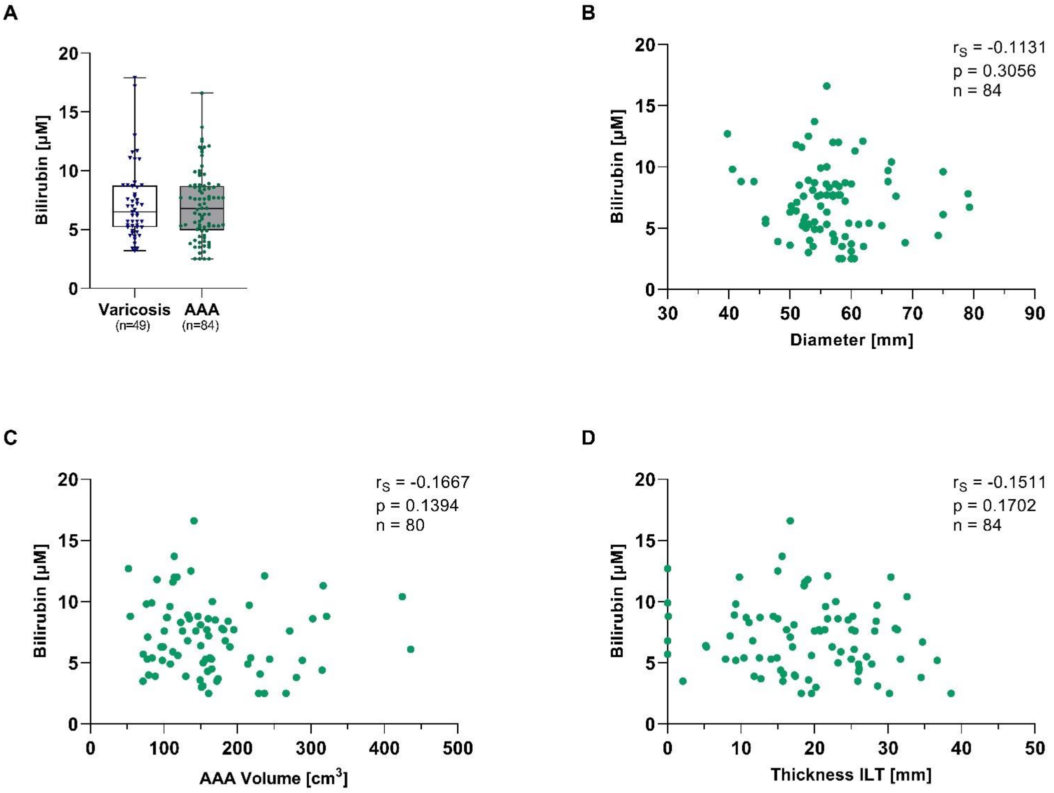

Supplementary Figure S3. Serum bilirubin concentrations and correlations with AAA diameter, AAA volume, and thickness of the intraluminal thrombus (ILT) in AAA and controls.

Total bilirubin was measured using standard laboratory methods at the Institute for Clinical Chemistry and Laboratory Medicine at the TU Dresden. **A,** Bilirubin concentrations were analyzed in electively treated AAA patients (AAA, green) and varicose controls (blue). **B-D,** Correlations of bilirubin with the AAA diameter, AAA volume, and thickness of ILT. Bilirubin was not measurable in 4 varicose vein patients and in 1 AAA patient. Data on AAA volume was missing in 4 AAA patients. **Statistics:** Significant outliers were identified by Grubb´s outlier test and excluded from further analysis. Data are shown as **A,** boxplots with individual values and analyzed by the Mann-Whitney U test. **B-C,** Spearman's correlation coefficient (r_S), and **D,** Pearson's correlation coefficient (r_P).

Supplementary Table S1. Primersequences

Gene	Forward (5′-3′)	Reverse (5′-3′)
**B2M**	GATGAGTATGCCTGCCGTGT	CATGATGCTGCTTACATGTCTCG
	NCBI Reference Sequence: NM_004048.4	
**TBP**	CGCCGGCTGTTTAACTTCG	AGAGCATCTCCAGCACACTC
	NCBI Reference Sequence: NM_003194.5	
**RPL32**	CACCGTCCCTTCTCTCTTCCT	TCTTGGGCTTCACAAGGGGT
	NCBI Reference Sequence: NM_000994.4	
**CD163**	GCGGGAGAGTGGAAGTGAAA	ACCTGCACTGGAATTAGCCC
	NCBI Reference Sequence: NM_001370145.1	
**NQO1**	CCCCGGACTGCACCAGAGC	CTGCAGCAGCCTCCTTCATGGC
	NCBI Reference Sequence: NM_001286137.1	
**Nrf2**	CCCAATTCAGCCAGCCCAGC	AACGGGAATGTGCTGCGCCAA

	NCBI Reference Sequence: NM_001313901.1	
**HMOX1**	AGTCTTCGCCCCTGTCTACT	CTTCACATAGCGCTGCATGG
	NCBI Reference Sequence: NM_002133.2	

Primers used for analysis of gene expression by quantitative real-time PCR (qPCR). **Abbreviations:** B2M–β-2-Microglobulin, HMOX1–heme oxygenase-1, NQO1–NAD(P)H quinone dehydrogenase 1, Nrf2–Nuclear Factor Erythroid 2-related Factor 2, RPL32–Ribosomal protein 32, TBP–TATA-box binding protein.

Supplementary Table S2. Plasma sCD163, cardiovascular risk factors, and medical therapy in AAA patients.

**Variable**	**Estimates**	**Variable**	**Estimates**
T2D treatment (ref=none)	1.39	Insulin (ref=none)	0.999
HDL cholesterol	1.356	Anticoagulation (ref=none)	0.991
β-blocker(ref=none)	1.178	Smoking (ref=none)	0.983
Triglycerides	1.137	Leukocytes	0.98
Hypertension (ref=none)	1.077	T2D (ref=none)	0.956
ARB (ref=none)	1.076	ACE (ref=none)	0.951
Statins (ref=none)	1.049	Hemoglobin	0.933
Diuretics (ref=none)	1.035	CAD (ref=none)	0.915
LDL cholesterol	1.028	CCB (ref=none)	0.839
Thickness ILT	1.007	PAD (ref=none)	0.812
CRP	1.005		

Plasma sCD163 was analyzed by ELISA, and the data were log transformed. A penalized (elastic net) linear regression was used to identify medical therapies and cardiovascular risk factors with a potentially stronger influence on plasma sCD163. Estimates show the increase or decrease in sCD163 when the patient is diagnosed with the indicated disease or receives the medical therapy compared to patients without (ref = none). For AAA diameter, thickness of ILT, LDL and HDL cholesterol, triglycerides, CRP, hemoglobin, and leukocytes, estimates refer to the increase or decrease per one unit. Treatment of type 2 diabetes (T2D) includes patients prescribed biguanides, sodium-glucose co-transporter-2 (SGLT2) inhibitors, glucagon-like peptide-1 receptor agonists (GLP-1-RA)/glucagon-like-peptide-1 receptor antagonists, dipeptidyl peptidase 4 (DPP-4)-inhibitors, and sulfonylureas. **Abbreviations**: ACE–angiotensin-converting enzyme, ARB–angiotensin II receptor blocker, ASA–acetylsalicylic acid, CAD–coronary artery disease, CCB–calcium channel blockers, CRP–C-reactive protein, HDL–high-density lipoprotein, ILT–intraluminal thrombus, LDL–low-density lipoprotein, PAD–peripheral artery disease, T2D–type 2 diabetes mellitus.

Supplementary Table S3. Serum bilirubin concentrations, cardiovascular risk factors, and medical treatment in AAA.

Variable	Estimate	Variable	Estimate
Insulin (ref=none)	1.853	CCB (ref=none)	0.978
Diuretics (ref=none)	1.409	T2D treatment (ref=none)	0.956
β-blocker (ref=none)	1.195	CAD (ref=none)	0.949
Hemoglobin	1.172	ACE (ref=none)	0.946
ARB (ref=none)	1.135	Leukocytes	0.94
Statins (ref=none)	1.006	PAD (ref=none)	0.934
T2D (ref=none)	1.006	Smoking (ref=none)	0.925
CRP	1	ASA (ref=none)	0.909
AAA diameter	0.999	Triglycerides	0.878
Thickness ILT	0.994	Hypertension (ref=none)	0.821
Anticoagulation (ref=none)	0.989	HDL	0.804
LDL	0.978		

Serum bilirubin was analyzed using standard laboratory methods at the Institute for Clinical Chemistry and Laboratory Medicine at the TU Dresden. A penalized (elastic net) linear regression was used to identify medical therapies and cardiovascular risk factors with a potentially stronger influence on serum bilirubin. Estimates show the increase or decrease in bilirubin when the patient is diagnosed with the indicated disease or receives the medical therapy compared to patients without (ref=none). For AAA diameter, thickness of ILT, LDL and HDL cholesterol, triglycerides, CRP, hemoglobin, and leukocytes, estimates refer to the increase or decrease per one unit. Treatment of type 2 diabetes (T2D) includes patients prescribed biguanides, sodium-glucose co-transporter-2 (SGLT2) inhibitors, glucagon-like peptide-1 receptor agonists (GLP-1-RA)/glucagon-like-peptide-1 receptor antagonists, dipeptidyl peptidase 4 (DPP-4)-inhibitors, and sulfonylureas. **Abbreviations**: ACE–angiotensin-converting enzyme, ARB–angiotensin II receptor blocker, ASA–acetylsalicylic acid, CAD–coronary artery disease, CCB–calcium channel blockers, CRP–C-reactive protein, HDL–high-density lipoprotein, ILT–intraluminal thrombus, LDL–low-density lipoprotein, PAD–peripheral artery disease, T2D–type 2 diabetes mellitus.

Supplementary Table S4: Serum bilirubin concentrations and associations with the AAA diameter.

Variable	Estimate	CI	*p*-value
AAA diameter	0.993	[0.980, 1.006]	0.294
HDL cholesterol	0.924	[0.663, 1.287]	0.640
Triglycerides	0.902	[0.796, 1.022]	0.110
Hypertension (ref=none)	0.802	[0.619, 1.039]	0.100
CAD (ref=none)	1.068	[0.875, 1.304]	0.519
T2D (ref=none)	0.830	[0.564, 1.222]	0.350
ACE (ref=none)	0.950	[0.747, 1.209]	0.680
ARB (ref=none)	1.148	[0.896, 1.471]	0.279
ASA (ref=none)	0.890	[0.715, 1.107]	0.298
β-blocker (ref=none)	1.165	[0.914, 1.486]	0.222
Anticoagulation (ref=none)	1.101	[0.804, 1.507]	0.552
Diuretics (ref=none)	1.300	[1.017, 1.66]	0.040
Insulin (ref=none)	1.779	[1.163, 2.723]	0.010

T2D treatment (ref=none)	1.082	[0.715, 1.637]	0.710

Serum total bilirubin concentrations were measured using standard laboratory methods at the Institute for Clinical Chemistry and Laboratory Medicine at the TU Dresden. Bilirubin was set as the outcome variable, and the effects of AAA diameter, HDL cholesterol, triglycerides, hypertension, CAD, T2D, ACE, ARB, ASA, β-blocker, anticoagulation, diuretics, insulin, and T2D treatment were tested by multivariate linear regression. Estimates are given on a log scale and show the increase or decrease in bilirubin when the patient has the indicated disease or receives the respective medical therapy (ref=none). For AAA diameter, thickness of ILT, HDL cholesterol, and triglyceride estimates, refer to the increase or decrease per one unit. Treatment of type 2 diabetes (T2D) includes patients prescribed biguanides, sodium-glucose co-transporter-2 (SGLT2) inhibitors, glucagon-like peptide-1 receptor agonists (GLP-1-RA)/glucagon-like-peptide-1 receptor antagonists, dipeptidyl peptidase 4 (DPP-4)-inhibitors, and sulfonylureas. **Abbreviations:** ACE–angiotensin-converting enzyme, ARB–angiotensin II receptor blocker, ASA–acetylsalicylic acid, CAD–coronary artery disease, HDL–high-density lipoprotein, ILT–intraluminal thrombus, T2D–type 2 diabetes mellitus.
